# How maize monoculture and increasing winter rainfall have brought the hibernating European hamster to the verge of extinction

**DOI:** 10.1038/srep25531

**Published:** 2016-05-06

**Authors:** Mathilde L. Tissier, Yves Handrich, Jean-Patrice Robin, Mathieu Weitten, Paul Pevet, Charlotte Kourkgy, Caroline Habold

**Affiliations:** 1 Université de Strasbourg, IPHC, 23 rue Becquerel 67087 Strasbourg, Cedex 2, France; 2 CNRS, UMR7178, 67087 Strasbourg, France; 3 CNRS, UMR7168, Institut des Neurosciences Cellulaires et Intégratives, Département de Neurobiologie des Rythmes, 5 rue Blaise Pascal, 67084 Strasbourg, Cedex, France; 4 Office National de la Chasse et de la Faune Sauvage, Au bord du Rhin, 67150 Gerstheim, France

## Abstract

Over the last decades, climate change and agricultural intensification have been identified as two major phenomena negatively affecting biodiversity. However, little is known about their effects on the life-history traits of hibernating species living in agro-ecosystems. The European hamster (*Cricetus cricetus*), once a common rodent on agricultural land, is now on the verge of extinction in France. Despite the implemented measures for its protection, populations are still in sharp decline but the reasons for it remain unclear. To investigate how environmental change has affected this hibernating rodent, we used a data set based on 1468 recordings of hamster body mass at emergence from hibernation from 1937 to 2014. We reveal the adverse effects of increasing winter rainfall and maize monoculture intensification on the body mass of wild hamsters. Given the links that exist between body mass, reproductive success and population dynamics in mammals, these results are of particular importance to understand the decline of this species. In view of the rates of maize monoculture intensification and the predicted increase in winter rainfall, it is of the utmost importance to improve land management in Western Europe to avoid the extinction of this species.

There is a consensus that agriculture and global warming are increasingly affecting wildlife^1^,^2^,^3^. Indeed, there has been growing evidence over the last two decades that climate change is affecting the demography and life-history traits of vertebrates^4^,^5^,^6^,^7^,^8^,^9^,^10^,^11^,^12^. Some species demonstrate a high phenotypic plasticity^6^,^8^, whilst others fail to adapt and consequently suffer a reduction in fitness^13^ and population decline^5^. However, climate change is just one of the numerous threats currently faced by wildlife, and species’ response to climate change depend on their distribution, their life-history strategies and whether or not they are affected by additional pressures such as pollution, fragmentation, invasive species or habitat loss^9^,^14^,^15^. Parallel to climate change, croplands and pastures have greatly expanded and now cover almost 40% of the land on Earth^1^. This phenomenon is associated with changes in agricultural practices^1^ and is currently supported through intensive cereal monocropping, mainly of maize^16^,^17^. The intensification of maize monoculture is known to cause soil degradation, the pollution of groundwater and biodiversity decline, affecting all species living in agricultural ecosystems^11^,^18^,^19^,^20^. Climate change and agriculture are directly (albeit partly) inter-related^1^: agricultural intensification is known to emit the greenhouse gases involved in climate change, which in turn directly affect agricultural production and sustainment. As a result, maize yields are expected to decrease by approximately 12% in the coming years^21^,^22^. Cumulatively, these elements directly alter the habitat of farmland species and make it less diversified and more unpredictable^16^,^23^. This stochasticity is even more damaging for small populations or species with a fast pace of life (*i.e*. a short lifespan and a high reproductive rate) and low dispersion capacities. Indeed, these animals are known to be less able to cope with “bad years”^24^ and are highly susceptible to the Allee effect, *i.e*. reduced fitness at low population density^25^,^26^,^27^. The few existing studies linking climate change, agricultural intensification and life-history traits of farmland vertebrates have been carried out on birds or on stable populations of badgers^11^,^28^. However, nothing is known to date about the effects of climate and agricultural changes on the life-history traits of hibernating species, nor have any studies investigated how these factors affect fast pace of life mammals living in agro-ecosystems. Here, we investigate for the first time the combined role played by climate and agricultural changes in the alarming decline of the European hamster (*Cricetus cricetus*), a hibernating rodent that is now endangered in almost all Eurasian range states and even locally extinct^29^,^30^. The extinction threat is greater in the Western-part of its distribution area (*i.e.* Netherlands, France, Germany and Belgium)^31^. It is widely recognized that peripheral populations (*i.e.* at the edge of the distribution) are genetically more differentiated than central ones, but are however facing a higher stochasticity in demographic processes^32^. Consequently, their conservation deserves high priority and might require specific measures^33^,^34^. However, it has been difficult to implement efficient conservation measures without a clear understanding of what causes the decline of the European hamster–especially in France, where it has been considered as a pest until the 90s^35^. During the two last decades, the focus has been on hamster population dynamics, and studies based on burrow surveys and genetics have highlighted the deleterious effects of insufficient protective cover on the mortality rate of wild hamsters^29^,^30^,^36^,^37^,^38^,^39^. It has recently been suggested that other factors could be involved in the decline of the species across Europe, including an effect of climate change on life-history traits or an overall decrease in reproductive success^35^,^40^. Yet nothing is known to date about the environmental factors that could affect the body condition, lifespan or reproductive success of wild individuals. In mammals, body mass–known to be related to fitness, predation risk and thus population dynamics^26^,^41^,^42^,^43^–is highly dependent on habitat suitability in agro-ecosystems^28^. In European hamsters, females only become fertile after the first winter if their body mass exceeds a threshold of 200 g^44^. Poor body condition at emergence may therefore greatly impair their reproductive success in spring, and consequently affect the population dynamic of the species. We thus used data recorded from 1937 to 2014 to investigate how climate change and agricultural modifications could have affected this species, focusing on the impact of these drivers on the body mass of wild individuals at the period of emergence from hibernation. We first looked at the trend in body mass and climate since 1937 and changes in crop diversity since 1989 in the French area of distribution of the species. We then focused on inter-annual fluctuations in body mass between 1992 and 2014 and looked for a correlation with variations in environmental parameters that could account for decreasing body condition. Finally, we focused on the impact of climate on body mass since 1937 to disentangle effects of temperature from those of rainfall on the trend observed in body mass.

## Results

### Trend in body mass change since 1937

**Figure 1:**
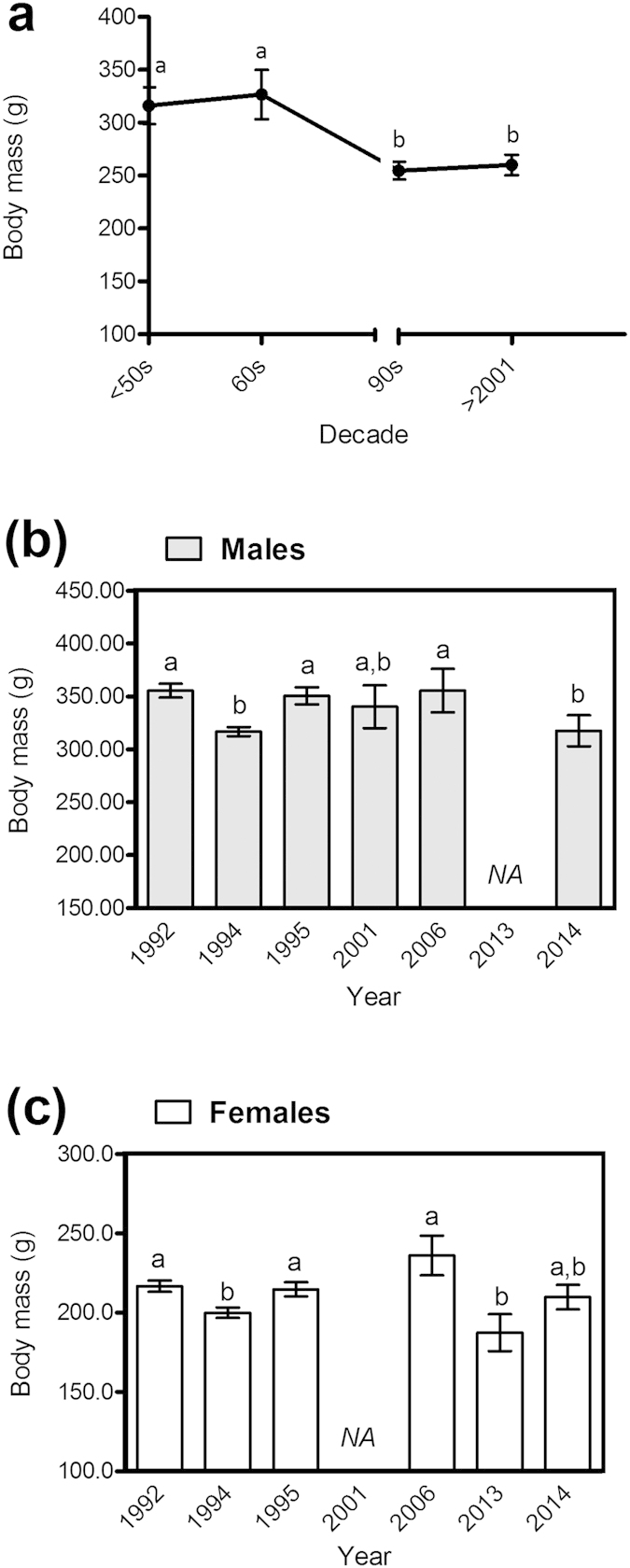
Change in body mass of wild hamsters (males and females) at the period of emergence from hibernation. (**a**) Body mass (g) is represented per decade from 1937 onwards (N = 1468; <50s corresponds to the period from 1937 to 1949, while >2001 represents the period from 2001 to 2014). Body mass (g) is represented per year since 1992 (**b**) in males (N = 720) and **(c)** in females (N = 672). Geometric means are represented ± SEM and different letters highlight significant differences (Multifactorial ANOVA, p < 0.05). See methodology section 3. for statistical details.

Hamster body mass at the period of emergence decreased by around 21% between 1937 and 2014 (Fig. 1a). Two plateaus can be observed: one showing a significantly higher body mass before the 70s, and the other from the 90s onwards, with significantly lower body mass (F_3,1467_ = 2.912, p = 0.033). Post-hoc analyses supported the findings in previous literature, showing that males are heavier than females, whatever the decade (384 ± 15 g and 259 ± 15 g respectively, F_2,1467_ = 668.2 and p < 0.001). Results also indicated a higher weight in May than in April (312 ± 7 g and 298 ± 8 g respectively, F_1,1426_ = 11.208, p = 0.001). We found no effect of an interaction between sex, month and decade on body mass (p > 0.2).

On the examination of changes in body mass from 1992 to 2014, we found significant variations between successive years, both in males and females (Fig. 1b, F_9,740_ = 3.348, p < 0.001 and Fig. 1c, F_5,672_ = 4.476, p < 0.001 respectively). Males showed significantly lower body mass at the period of emergence in 1994 and 2014 (Fig. 1b; data unavailable for males in 2013). Females were significantly lighter in 1994 (Fig. 1c) and 2013 (Fig. 1c), but they did not show any sign of weight loss in 2014.

### Changes in environmental variables and impact on body mass

**Figure 2:**
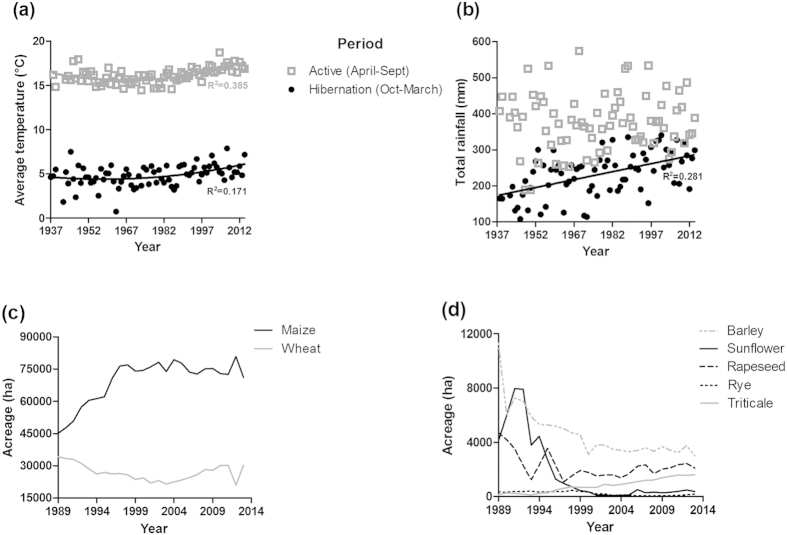
Inter-annual change in temperature, rainfall and crop acreage in the Bas-Rhin (Northern Alsace, France) since 1937 (climate change) and 1989 (crop acreage). (**a**) Average temperature (°C) and (**b**) total rainfall (mm) are shown according to the year and the period of the biological cycle of the common hamster (active period and hibernation). (**c**) Crop acreage (ha) of the main cereals (wheat and corn) and of (**d**) five other crops (rapeseed, barley, rye, sunflower and triticale) according to the year.

When looking at inter-annual climate variation since 1937, we observed a slight increase in average temperatures during hibernation (October-March) and the active period (April-September) of the species (Fig. 2a, R^2^ = 0.171, p < 0.001 and R^2^ = 0.385, p < 0.001, respectively). Although there was no variation in the average annual rainfall, a significant increase was observed in rainfall averages during hibernation (Fig. 2b, R^2^ = 0.272, p < 0.001). Changes in the acreage for different crop types were due to the expansion of maize and triticale crops from 1989 onwards (57% and 1233% respectively, Fig. 2c,d), whilst the amount of land used for other crops such as wheat, rapeseed, barley, rye and sunflower decreased by 12%, 56%, 74%, 49% and 91% respectively (Fig. 2c,d).

**Figure 3:**
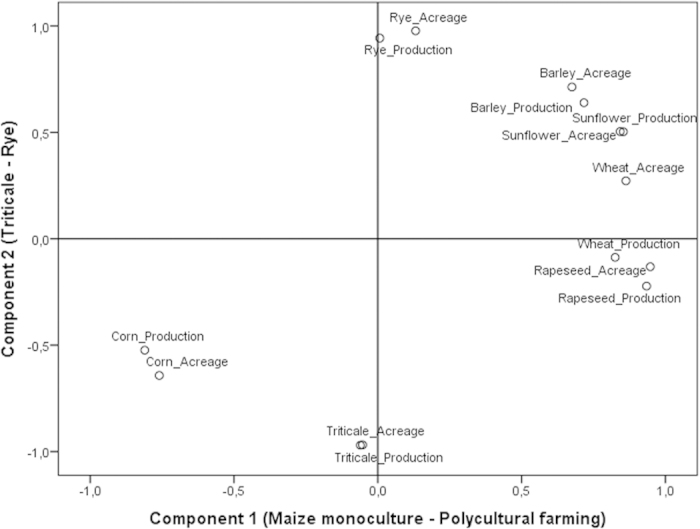
Factorial map of PCA analysis on agriculture variables. Variables include production (per 100 Kg) and acreage (in ha) of seven crops (rye, corn, triticale, barley, sunflower, wheat and rapeseed). The component 1 axis opposes maize monoculture (<−0.5) to polycultural farming (>0.5), while the component 2 axis mainly opposes triticale and rye crops. See methodology section 3. for statistical details.

To investigate the effects of agricultural change on body mass, we first carried out PCA analysis of fourteen agricultural variables (surface and production of seven crops; see methodology for details); (Fig. 3). The PCA extracted two components which explained 91.1% of the total variance: the first opposed maize monoculture (negative values) to polycultural farming (*i.e*. wheat, rye, sunflower, rapeseed and barley; positive values, Fig. 3, X axis). The second component mainly opposed two cultures: rye (positive values) and triticale (negative values, Fig. 3, Y axis).

**Figure 4:**
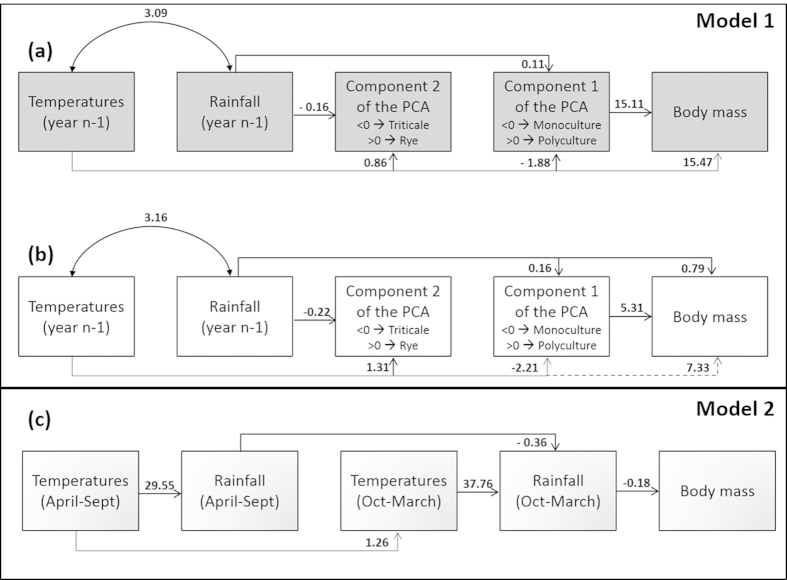
Path analysis diagrams showing the impact of climate and agriculture (PCA components) on the body mass of wild hamsters. Model 1 represents multiple relationships between temperatures, rainfall, agricultural variables and body mass of (**a**) males and (**b**) females from 1992 to 2014, and model 2 (**c**) shows multiple relationships between climatic variables and body mass of males and females from 1937 to 2014. Arrows indicate significant directed links between variables. Unstandardized estimates (which can be positive or negative) are indicated along their respective paths. Significant paths are indicated by solid arrows while the dashed arrow represents a non-significant path remaining in the selected model (0.05 < p < 0.1). See methodology for statistical details.

Secondly, we used path analysis to see how agriculture (components of the PCA) and climate (temperature and rainfall) affected the body mass of hamsters at emergence from hibernation (see methodology for details). The first path analysis (Model 1, Fig. 4a,b) revealed that body mass (from 1992 onwards) was strongly and positively related to polycultural farming (wheat, rye, sunflower, rapeseed and barley) and therefore negatively related to maize monoculture in both sexes (Fig. 4a,b, p < 0.05). We did not find any significant relationships between component 2 of the PCA and body mass in males or in females (Fig. 4a,b, p > 0.1). Temperatures and rainfall had differential effects on body mass, depending on the sex. Whilst male body mass at emergence was strongly and positively related to average temperature in year n-1, it did not seem to be affected by annual rainfall (Fig. 4a, p = 0.01 and p > 0.1 respectively). Conversely, female body mass was positively related to annual rainfall (year n-1), but not to average temperature (Fig. 4b, p = 0.031 and p > 0.1 respectively).

The second path analysis (Model 2, Fig. 4c) concerned the impact of inter-annual change in climate on body mass at the period of emergence (from 1937 onwards), and revealed that this trait was significantly and negatively related to rainfall during hibernation in both sexes (Fig. 4c, p < 0.009). This was the only direct link found between climatic variables and body mass in males or females (Fig. 4c, p > 0.09).

## Discussion

The European hamster is a species with a fast pace of life, *i.e.* a short lifespan and a high reproductive rate that should compensate for a high predation rate^27^,^44^. It is widely recognized that introduction effort and the size and number of litters are the most important parameters for mammal population growth from small numbers^27^. Yet despite the theoretical high reproductive rate of these mammals and the strong reinforcement measures applied to protect these populations, the species has shown a decrease of 94% in its French distribution area since 1972^30^. Interestingly however, genetic diversity has been preserved^39^. Given the importance of genetic diversity of margin populations for the long-term conservation of species^32^, French populations thus justify a high conservation priority. To reverse the decline and enhance the overall viability of these local populations, the French government has launched successive Conservation Plans for the period 2000–2016^31^. These plans include reinforcement operations and the reconstruction of a network of alfalfa or wheat plots covering several acres, which are partially harvested. Although these measures have helped to slow down the decline, they do not appear to be sufficient to increase the hamster population. The evaluation report concerning these action plans underlines the serious lack of knowledge about how multiple threats affect the biology and demographic parameters of hamster populations. Our study reveals that climate change and maize monoculture have played a combined role in reducing the body mass of wild hamsters by up to 21% since 1937. Given the existing links between body mass, predation risks and reproductive success^26^,^41^,^42^,^43^, it is very likely that this high decrease in body mass has affected the reproduction and life expectancy of hamsters. Increased predation rates^29^,^36^,^45^ and reduced life expectancy (from 4 to 2 years^29^,^44^) have been recently reported in this species. Given that individuals gain mass throughout their life^44^, this decrease in the average age of wild populations could also partly explain the decline in body mass that we observed. Our study sheds new light on how environmental change might have affected reproduction, a subject that was little understood until now^40^. We found that female body mass at emergence reached extremely low values in particular years (*e.g*. 186.47 ± 13.2 grs in 2013). As we indicated earlier, female European hamsters only become fertile after the first winter if their body mass exceeds a threshold of 200 g^44^. This overall decrease in body mass could therefore greatly impair the reproductive success of females by delaying the first reproductive event and reducing the number of females that produce a litter. This is supported by recent unpublished data on the reproductive success of the species in France^46^. Litter size-recorded as 5–8 pups per litter across Europe^44^,^47^-is currently estimated at 2.4 ± 1.6 pups in France^46^. More importantly, litter number, recorded as 2–3 litters/female/year^44^,^47^ is currently less than 1 litter/female/year in France^46^.

The affirmation that intensive agriculture is the main cause in the decline of the European hamster has recently been questioned, and global warming has been proposed as another cause of hamster population decline in Western Europe^40^. In our analysis, Model 1 revealed a strong negative effect of maize monoculture (which has increased by 57% since 1989) over the last two decades (β = 15.11, Component 1 of the PCA), and Model 2 revealed that from 1937 to 2014, winter rainfall (which had increased by 28% during this period) had a slightly negative effect on body mass (β = −0.18), which had declined by 21% during the same period.

Changes incurred by maize are thus relatively recent (with an intensification at the end of the 80s) and seem to have had a strong effect on body mass over a relatively short timescale. Conversely, winter rainfall started to increase at an earlier date (1937), and its effect on body mass seems to be less abrupt but to have had an effect over a longer period of time. These links between environmental variables and hamsters’ body mass can be illustrated by two extreme examples. First, the 2012 explosion in maize production (following an extremely cold winter in 2011–2012 that damaged wheat production) was followed by a significantly reduced female body mass at emergence in 2013, associated with a high decrease in the number of burrows the same year^48^. This higher production of maize–associated with an increased allocation of land to this cereal–implies that less agricultural land was used for other crops. This might ultimately lead to less varied food resources for hamsters during the winter hibernation period. Given the importance of food stores for this species^44^ and the poor nutritional quality of maize^49^, this could negatively affect hibernation quality and thus hamster body mass at emergence. Moreover, as the stubble is removed from maize fields after harvest in France, the microclimate (*i.e*. ground temperature and humidity) in these bare plots during winter might be different to that found in wheat plots, which remain covered by crops throughout the winter^50^. Given that hibernation is known to be optimal at a given temperature^51^, animals in these plots may increase their energy expenditure, leading to greater loss of body mass. These non-mutually exclusive hypotheses could explain the decline in body mass that we observed in animals emerging after years with high maize production. These ideas are supported by the observation that hamsters emerging in a field of maize ultimately leave the plot in the spring. However, they might remain on the maize plot if it had been previously seeded with cereals (*personal com*.). In Germany (where maize occurrence ranges from 12% to 19%, compared to 55 to 80% in Alsace, France), it has been demonstrated that the occurrence of hamsters decreases as the presence of maize increases, with no hamsters found in the areas where more than 18% of land was covered by maize^52^. Researchers in the Netherlands do not prospect in maize fields that they consider as an unfavourable crop for the species^29^. The second example of links between environmental variables and hamster body mass is the negative link between winter rainfall and body mass, illustrated by the reduced body mass of males emerging after the particularly warm and wet winter in 2013–2014 (*i.e*. 44% more rainfall than in 2006). In contrast to studies on non-hibernating vertebrates highlighting that increasing temperatures have an effect on a variety of species^4^,^5^,^6^,^28^, our study reveals that European hamsters seem to be more affected by increasing winter rainfall than by temperatures. However, our model revealed that increasing winter rainfall is associated with a warmer climate, which thus indirectly affects hamster body mass. This finding echoes recent studies in hibernating mammals stating that winter conditions (*i.e.* winter Pacific Decadal Oscillation) and warmer climates can negatively affect the duration of hibernation and the survival of individuals^53^,^54^. We can imagine that increasing winter rainfall would increase soil moisture in depth (by percolation and rising of groundwater, located two to three meters underground in Alsace). During winter, hamsters live in a ~2 metre-deep burrow^44^ and would thus probably experience changes in soil moisture more than changes in above-ground temperatures. Increased rainfall could lead to higher levels of soil moisture during hibernation and wet the animal’s fur, ultimately reducing the insulation it provides and increasing the cost of hibernation through higher heat loss and greater energy expenditure, inducing faster body-mass loss. Another hypothesis is that wet soils could lead to the fast deterioration of food hoarded in the burrow, resulting in lower stores of intact food and/or a lower quality of available food reserves for the winter. A study in kangaroo rats^55^ has shown that individuals can remodel their burrow and increase the number of exits in response to high rainfall, presumably to increase the evaporation rate and avoid the deterioration of seed caches. No study to date describes precisely how European hamsters manage the different stocks of food inside their burrow, *i.e.* whether the entire stock is stored at the same place and depth and if it is therefore equally affected by soil moisture. The effect of winter rainfall on body mass at emergence could be of particular importance to start disentangling the reasons behind the decline of Central populations of the species (*i.e*. in Eastern Europe, where maize monoculture has not expanded to the same extent as in Western Europe). This idea is supported by a recent study highlighting that the current shrinkage of the species range in Europe may be a response to the oceanic climate gradient extending eastwards in Europe^56^. Regarding the negative impact of maize monoculture on body mass at emergence, our results suggest that it could have played a major role in the decline of the European hamster in France, covering 55 to 80% of its natural habitat. However, the multiple relationships presented above do not allow us to determine whether it is the maize itself (*i.e.* its composition), monoculture (*i.e.* a lack of food diversity/availability or a particular micro-climate) or both of these elements combined that negatively affect body mass at emergence. We will thus further investigate the nutritional value of maize for this species, and in parallel, look at how the fitness of wild individuals is affected by maize monoculture in Alsace.

Our results ultimately suggest that the overall decrease observed in body mass over the last century might have affected the reproduction and survival of this species, and thus population dynamics. A recent study in the European badger revealed positive links between global warming, increasing quality of habitat in agricultural lands, body weight and population size in this species^28^. Although the direction of the link is in the direct opposite of what we observed in the European hamster, the links between environmental parameters, body mass and population dynamics are consistent. Our results also provide objective knowledge that is underpinning concrete management proposals for the sustainment of European hamster populations in France. Further studies are needed to extend our understanding of the underlying mechanisms that explain the impact of maize or winter rainfall on the fitness of this species. Given the high rates of maize monoculture intensification and climate change, we can expect the decline of European hamster populations to continue. This is especially true if nothing is done to improve land management and inverse the predominance of intensive maize monoculture in Western Europe. One solution would be to manage agricultural landscapes by managing field plots with a combination of sharing and sparing strategies^57^ in order to improve soil quality, the abundance of invertebrates and thus habitat suitability for the European hamster.

## Material and Methods

### Species and study site descriptions

The European hamster lives in loose and stable soils, which are also particularly adapted to crops. Individuals have a marked seasonal body mass cycle in which they gain weight in summer^44^. On average, males are longer and larger than females (27–32 cm and 350 g, and 22–25 cm and 260 g, respectively). Hamsters are ‘food-storing’ hibernators: they store large amounts of food in their burrow before hibernating^44^ and feed on these stocks during their winter arousals. Hibernation lasts from late September to April, with variations occurring according to sex, age and body condition^38^,^44^.

In France, the current relict population of the species (<1500 individuals) is solely found in the agricultural ecosystems of the Alsace plain^30^,^39^, dominated by maize monoculture (that covers up to 80% of all cropland). In this study, we focused on a “central” population representing 80% of total European hamster population in Alsace^39^, located in the vicinity of Blaesheim (Alsace, France, N48°30′14.044” and E7°36′28.414, elevation: 154 m). The average size of agricultural plots in this area has tripled since 1971 (1.4 ha in 2010, compared to 0.54 ha in 1971). This surface area is equivalent to the home range of a male, and is seven times the size of the home range for a female^38^.

### Trend in body mass change since 1937

We used individual body mass data collected between 1937 and 2014 by CNRS research teams (C. Kayser, B. Canguilhem, A. Malan and P. Pévet) and by the National Hunting and Wildlife Agency (ONCFS). This experiment was in accordance with EU 270 Directive 2010/63/EU guidelines for animal experiments and the care and use of laboratory 271 and wild animals. It was approved by the Ethical Committee (CREMEAS) under agreement 272 numbers 00624-01 and 00305-01. Wild hamsters were captured and weighed from 1937 to the 60s by CNRS research teams (C. Kayser, B. Canguilhem and A. Malan) for captive studies on hibernation. During the 90s, P. Pévet (CNRS) captured, weighed and then used hamsters for the establishment of a breeding unit and studies on biological rhythms. Finally, from 2001 to 2014, the ONCFS captured and weighed individuals before releasing them immediately. Data concerning the reproductive success of wild females have only been available since 2014. The different teams followed the same protocol for capture (always starting from the 1–5^th^ of April and ending on 30^th^ of May) and body mass measurement. We focused on body mass in April and May as a proxy measure to evaluate the body condition of individuals emerging from hibernation, as body mass at this period is a key factor in survival and reproductive performances^44^. Data included body mass at the period of emergence for 1468 individuals: 660 females, 742 males and 66 individuals for which the sex was unknown. Analyses were run both with and without these 66 individuals, but since the trend was equivalent in both cases, we retained data for these individuals in our analysis. Data were spread over 19 years between 1937 and 2014 and computed to period class (*i.e*. “decades”): <50s (up to and including 1949), the 60s, the 90s and 2001–2014. We tested the possible impact of (i) sex, (ii) decade, (iii) month (April or May) and interactions between (iv) sex*decade and (v) sex*month on body mass.

We then focused on the inter-annual fluctuations of body mass between the early 90s and 2014, the period for which we had the most detailed data set. This enabled us to look at the impact of environmental variables on this trait while excluding confounding factors such as hunting (the species has been protected since the early 90s but was previously actively trapped and poisoned^30^,^35^,^39^). Analyses were run separately for each sex due to the sexual dimorphism in this species and because data was missing for males (2013) and females (2001). We then tested whether (i) year and (ii) month (April or May) had an impact on body mass.

### Changes in environmental variables and impact on body mass

To investigate the impact of environmental change on body mass from 1992 to 2014, we focused on the relationships between body mass at emergence (year n), climate (year n-1) and crop diversity/availability (year n-1). We first looked at how inter-relations between rainfall, temperatures and agriculture (year n-1) could impair body mass at emergence (year n). The climatic parameter was composed of (i) average annual temperature and (ii) total annual rainfall (data from Météo France, Entzheim station). The agricultural parameter was derived from data on the production (per 100 Kg) and acreage (ha) of seven crops: wheat (*Triticum aestivum*), maize (*Zea mays*), sunflower (*Helianthus annuus*), barley (*Hordeum vulgare*), rye (*Secale cereal*), triticale (*Triticosecale sp*.) and rapeseed (*Brassica napus*).

In a second analysis (Model 2), we tried to understand whether body mass on emergence (year n) had been more affected by the climate during the winter (rainfall and temperatures from October to March, year n-1 to year n) or by the climate during the active period (from April to September, year n-1) from 1937 to 2014. The climatic parameter was composed of (i) the average temperature and (i_2_) the total rainfall during the active period, and (ii) average temperature and (ii_2_) total rainfall during hibernation.

### Statistical analyses

Data for body mass change were analysed using multifactorial ANOVAs. Normality was tested using a Kolmogorov-Smirnov test and variance homogeneity was checked using a non-parametric Levene test. Body mass variables were log-transformed to fulfil normality conditions. Multiple comparisons were analysed via post-hoc LSD testing. We back-transformed the data using the antilog^58^ for representation of body mass (rather than Log_10_ of body mass) in Fig. 1: data represented are geometric means ± SEM.

Data for climate change (temperature and rainfall) were analysed using quadratic and linear regressions, respectively. We carried out a principal component analysis (PCA) to reduce the number of agricultural variables and to extract the main ones.

Path analysis^59^ was then used to test for the presence, nature and strength of multiple relationships between environmental variables (rainfall, temperature, components of the PCA) and body mass. The most parsimonious model was selected by removing insignificant paths one by one. We only removed a path if the Chi-square (which tests the null-hypothesis that the reduced model fits the data as well as the saturated model) value of the model remained >0.1. Analyses were conducted using IBM SPSS software (IBM Corp. Released 2012. IBM SPSS Statistics for Windows, Version 21.0. Armonk, NY: IBM Corp; SPSS-AMOS for path analysis), and the significance threshold was set at p < 0.05.

## Additional Information

**How to cite this article**: Tissier, M. L. *et al.* How maize monoculture and increasing winter rainfall have brought the hibernating European hamster to the verge of extinction. *Sci. Rep.* **6**, 25531; doi: 10.1038/srep25531 (2016).
